# Social hierarchy is established and maintained with distinct acts of aggression in male *Drosophila* *melanogaster*

**DOI:** 10.1242/jeb.232439

**Published:** 2020-12-23

**Authors:** Jasper C. Simon, Ulrike Heberlein

**Affiliations:** Janelia Research Campus, HHMI, Ashburn, VA 20147, USA

**Keywords:** Dominance, Behavioral sequence, Escalation, Fighting, Genetic mutants, Fruit fly

## Abstract

Social interactions pivot on an animal's experiences, internal states and feedback from others. This complexity drives the need for precise descriptions of behavior to dissect the fine detail of its genetic and neural circuit bases. In laboratory assays, male *Drosophila melanogaster* reliably exhibit aggression, and its extent is generally measured by scoring lunges, a feature of aggression in which one male quickly thrusts onto his opponent. Here, we introduce an explicit approach to identify both the onset and reversals in hierarchical status between opponents and observe that distinct aggressive acts reproducibly precede, concur or follow the establishment of dominance. We find that lunges are insufficient for establishing dominance. Rather, lunges appear to reflect the dominant state of a male and help in maintaining his social status. Lastly, we characterize the recurring and escalating structure of aggression that emerges through subsequent reversals in dominance. Collectively, this work provides a framework for studying the complexity of agonistic interactions in male flies, enabling its neurogenetic basis to be understood with precision.

## Abstract (precis)

**Summary:** Precise descriptions of aggression aligned to changes in social status reveal how the recurring sequence and escalation of distinct behaviors relate to the establishment, maintenance and reversal of social dominance.

## INTRODUCTION

Agonistic interactions essential for establishing social hierarchies over food, territory or mates often progress as a sequence of stereotyped acts. Like other goal-driven behaviors (Berridge, 2004), the steps along these sequences are triggered by context and modified by past experience (Freudenberg et al., 2015; Kravitz and Huber, 2003), through largely unknown mechanisms that are coupled to an animal's internal state (Anderson, 2016). Determining these mechanisms remains difficult, however, because social exchanges proceed as a dynamic, reciprocal and continual feedback loop of interactions between two (or more) decision-making individuals that progresses over both moment-by-moment and protracted time scales (Chen and Hong, 2018). Inter-male adversarial contests in the fruit fly *Drosophila melanogaster* are well suited for studying such complex social phenotypes, owing to their established study in a laboratory setting (Baier et al., 2002; Chen et al., 2002) and the multitude of techniques available for manipulating and recording gene and neuron function (Sherer and Certel, 2019). Nonetheless, efforts to explain which behaviors lead to dominance hierarchies (Vrontou et al., 2006; Yurkovic et al., 2006) have suffered from a lack of consensus on methods and results for studies of aggression (as discussed within Asahina, 2017; Kravitz and Fernandez, 2015).

Here, we used an explicit criterion to characterize the temporal relationship among various aggressive acts and the establishment and reversals in dominant hierarchical status between pairs of male flies in arenas commonly used for high-throughput phenotyping (Dankert et al., 2009; Dierick, 2007). Using this approach, we observed that the acts of fen­cing, boxing and tussling appear well positioned in time to be related to the establishment of social dominance, whereas lunging and chasing appear related to its maintenance. We verified that lunging, the most frequently used behavior for quantifying aggression, unexpectedly occurs nearly exclusively after the onset of dominance, and is therefore unlikely to be involved in its establishment. In fights between high- versus low-lunging genetic lines, males became dominant at chance levels. This result suggests that the total number of lunges executed by an individual is insufficient for explaining his social outcome. We further observed that dominant males continue lunging when combating familiar opponents, and also when they confront unfamiliar opponents in novel settings. Lastly, we report that a fraction of the aggressive acts surveyed intensified through subsequent reversals in social dominance, adding to a fuller appreciation of this complex, recurrent social exchange. Together, this work provides a framework for untangling which aggressive acts causally relate to the establishment of social hierarchy and those that are a consequence.

## MATERIALS AND METHODS

### Fly lines

All *Drosophila* *melanogaster* Meigen 1830 lines originate from a P-element collection generated by the Heberlein laboratory that were then backcrossed for five generations to *w^1118^* Berlin.

### Animal rearing

To lessen developmental heterogeneity, males used in experiments were collected from lines maintained with controlled densities (by seeding vials with five males crossed to 10 females and removing these parental animals after 3 days), reared in customary 8-dram plastic vials on standard medium (cornmeal/yeast/molasses/agar), maintained at 25°C and 65% relative humidity, and entrained on a 12 h:12 h light:dark cycle. The lights-on phase started at 13:00 h EST. Transitions between dark and light were immediate.

### Animal handling

To model a uniform, probable ‘ecological baseline’ amount of social experience, including prior mating, males for experiments were collected after 7 h, yet within the first 24 h following eclosion using CO_2_. For experiments in which the identity of individuals was required, during collection, a small section of wing chosen randomly from one of an eventual pair was removed, a procedure shown to be inconsequential to the outcomes of adversarial contests (Kim et al., 2018).

### Animal housing

In order to increase or suppress the level of aggression, males were housed individually in 10-mm diameter×75-mm tall glass tubes (Fisher Scientific, Waltham, MA, USA) or as groups of 15 males in 8-dram vials (Hoffmann, 1990; Wang et al., 2008). For both housing conditions, flies had *ad libitum* access to standard medium and were incubated in the same conditions as they were reared.

### Animal observation

Experiments were performed on 4- to 6-day-old males that were removed from food immediately prior to start. Trials began after a 30-min adjustment period to ‘lights on’ and the environmental conditions of the observation room. All runs were completed within the first 4 h of ‘day’. Replicate experimental and control trials were intercalated and duplicate batches of trials were run over several days at least twice, weeks to months apart.

### Aggression assay

Unless noted, aggression assays were performed similarly to those previously described (Hoopfer et al., 2015) modified from Dierick (2007). Briefly, pairs of males were aspirated simultaneously into a 16-mm diameter×10-mm tall enclosed arena staged in an environmentally controlled room held at 25°C and 45% relative humidity and their behavior was recorded for 20 min. To encourage consistent aggressive interactions, the entire floor of the arena was composed of a thin layer of apple juice–sugar agar made as 10 g sucrose and 9 g agar boiled in 400 ml 100% apple juice (Mott's, Plano, TX, USA). To keep the quality of the agar floor consistent, it was used either immediately after a 2-h setting period or air-dried for 1 h, wrapped inside of plastic (Saran, Racine, WI, USA), and held at room temperature until the following day. To impede flies from climbing, the wall of the arena was made slippery by coating it with Fluon (BioQuip, Rancho Dominguez, CA, USA). Similarly, to limit flies from hanging from the ceiling, the lid of the arena was brushed with Sigmacote (Sigma-Aldrich, St Louis, MO, USA), a transparent silicone paint allowing an unobstructed view for recording from above. Both coatings were left to dry for at least 24 h before running experiments.

### Aggression screen

The genetically high- and low-lunging males used came from a P-element screen (Mark Eddison, J.C.S, U.H., unpublished; see Fig. S1A for the distribution of median total lunges for all lines observed in screen). From this screen, only lines that maintained a stable phenotype after backcrossing, had normative levels of activity (as estimated by measuring their total distance traveled and total number of jumps) and appeared otherwise healthy were used. A line, 5.116, exhibiting normative levels of aggression and activity was also identified in the screen. This ‘standard’ was used for all principal experiments reported within this work. Table S1 includes measures of the aggressive acts from the various lines used within the study.

### Data acquisition

Interactions between males were captured at 30 frames s^−1^ using a Basler A622f camera (Edmund Optics, Barrington, NJ, USA). Recordings from an array of arenas were cropped into 144×144 pixel resolution movies for each arena containing a pair of males and saved individually at 83.4 dots cm^–1^ with gVision video acquisition software (http://gvision-hhmi.sourceforge.net) for further analysis. Experimental arenas were lit from behind with a flicker-free, uniform, white backlight (Coherent, Santa Clara, CA, USA), making recordings suitable for machine vision tracking and behavioral classification methods. For the principal experiments reported in this work, recordings started within 2 min after introduction. For experiments analyzed from the P-element screen, recordings started 5–7 min after introductions, allowing sufficient time for loading and 5 min for flies to settle.

### Behavior classification

To catalog the changes in social hierarchy and also the aggressive acts, we used a combination of manual and automatic classification methods (Fig. S2A,B and Table 1 for methods, descriptions and software used). In summary, the establishment and reversals of social dominance and also the aggressive acts – fence, box and tussle – were manually annotated with VCode (Haegorn et al., 2008) and we used CADABRA (Dankert et al., 2009) to automatically classify wing flick, lunge and chase. To assign specific lunges to identified males, we used a semi-automated method previously reported (Kim et al., 2018). This software application uses as inputs both original full-length movies and corresponding time-stamped behavioral events (generated with CADABRA in our case; Dankert et al., 2009), and iteratively makes short movies inclusive of each event, allowing users to accurately assess whether a lunge was executed or which male executed a particular lunge. For the P-element screen, pairs of males were tracked and automatically scored for total number of wing flicks, tussles, lunges, chases and jumps using CADABRA (Dankert et al., 2009).

**Table 1.**
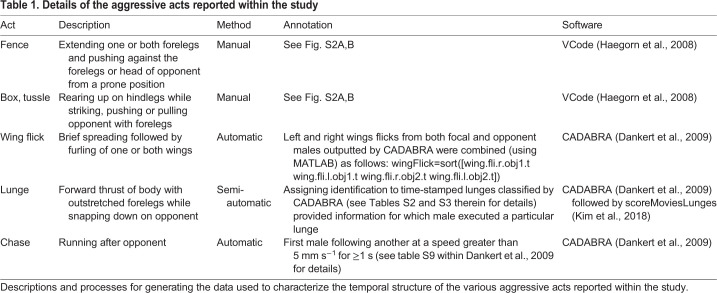
**Details of the aggressive acts reported within the study**

### Statistical analysis

Data processing, plotting and statistical analyses were all conducted in MATLAB (MathWorks, Natick, MA, USA). Details for analyses are explained and reported within each figure legend. See Fig. S2C for specifics regarding data exclusion.

### Code availability

Documentation and code used for verifying the identification of which male lunged are available at: https://github.com/JasperCSimon/scoresMovie.git.

## RESULTS

### Identifying hierarchical relationships

**Fig. 1.**
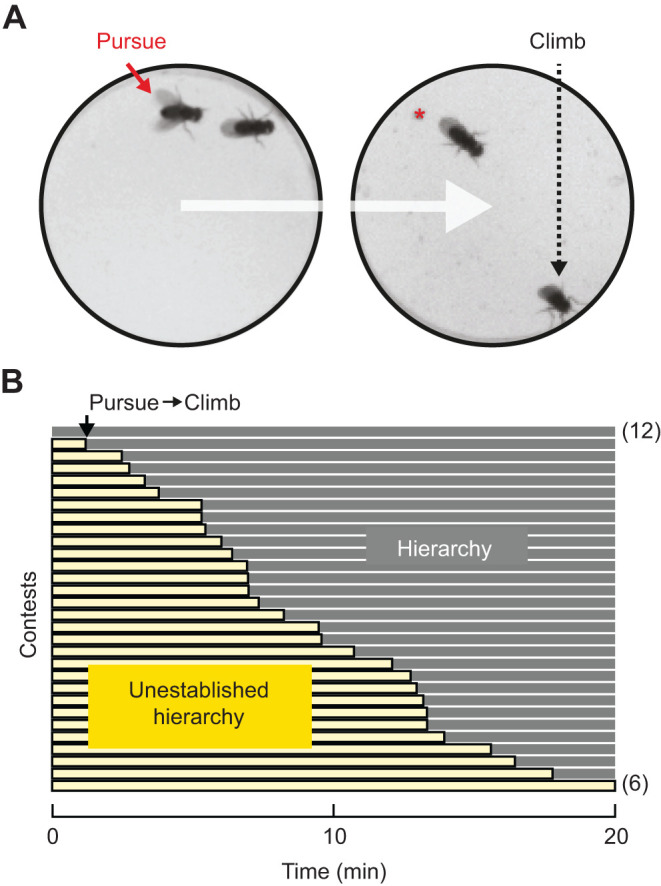
**Identifying the onset of social hierarchy within arenas used for studies of aggression.** (A) Example movie images illustrating the diagnostic ‘pursue-to-climb’ criterion for identifying the onset of social hierarchy. Pursuing males (red solid arrow), that often proceed to guarding the floor (red asterisk), are classified as dominant and those then attempting to flee by climbing the wall of the arena as subordinate (black dashed arrow). (B) Twenty-minute contests ordered as rows from early to late onset of establishment (top to bottom). Using the diagnostic criterion, clear hierarchies form in the majority of pairings. Periods of unestablished social standing (light yellow bars) precede clear social hierarchy (gray bars). Contests in which hierarchies formed prior to observation (full-length gray bar, top row, *n*=12) and those in which hierarchies never formed (full-length yellow bar, bottom row, *n*=6) did not change results and were excluded from further analysis.

Upon introduction to experimental arenas, pairs of males display independent and haphazard movements that typically include one or both males jumping, taking flight and/or climbing the wall as they attempt to escape the arena. With time, the males settle into a period of exploration, during which they eventually meet. This initial contact ends their independent movements and leads to bouts of short, alternating pursuits that are characteristically restricted to the floor, which was entirely composed of food (see Materials and Methods). This engagement then predictably progresses to a situation wherein one male consistently pursues the other until the retreating male leaves the floor, presumably in an attempt to flee by climbing the wall. It is this diagnostic ‘pursue-to-climb’ sequence that we used as a criterion to identify the initial establishment of a hierarchical relationship, with the pursuing male being classified as dominant (Fig. 1, Movie 1). This diagnostic criterion is also used to identify reversals in social status, as discussed later within this work.

### Distinct aggressive acts precede, concur or follow the establishment of dominance

We observed adversarial contests in experimental arenas that have been widely used to study aggression (Dankert et al., 2009; Dierick, 2007). These arenas, which have a floor comprised entirely of food, increase the frequency of flies' interactions by providing constant access to a food resource and yet retaining an uncluttered background with no food cup and no decapitated female (Certel and Kravitz, 2012); moreover, the entirety of the chamber is within view of the camera. This arrangement permits use of computer vision methods to help automatically annotate various aggressive acts over time (Dankert et al., 2009), followed by direct manual inspection for correcting erroneous annotations and identifying the onset of dominance. Together, this complementary workflow allows for a robust, high-resolution portrayal of the aggressive acts displayed during the progression of agonistic interactions.

Males who had never experienced a fight (hereafter, ‘naive males’) were introduced together as pairs into arenas. This simultaneous introduction lessened the disparity in which individuals discovered the contested resource and allowed uninterrupted observation of their interactions leading up to the onset of social dominance. Over a period of 20 min, we measured the occurrence of several acts, including wing flicks and the following acts reported to span the range of aggression, listed here from low to high intensity: fencing, chasing, lunging, boxing and tussling (Chen et al., 2002). Note that chasing is a long, high-speed trailing behavior that is distinct from pursuits, which are shorter. At times we found boxing and tussling difficult to distinguish, and thus for ease of scoring they were combined into the single category, ‘box, tussle’, as done by others (Chen et al., 2002). See Fig. S2A,B and Table 1 for details of behavioral classification, annotation and software used.

**Fig. 2.**
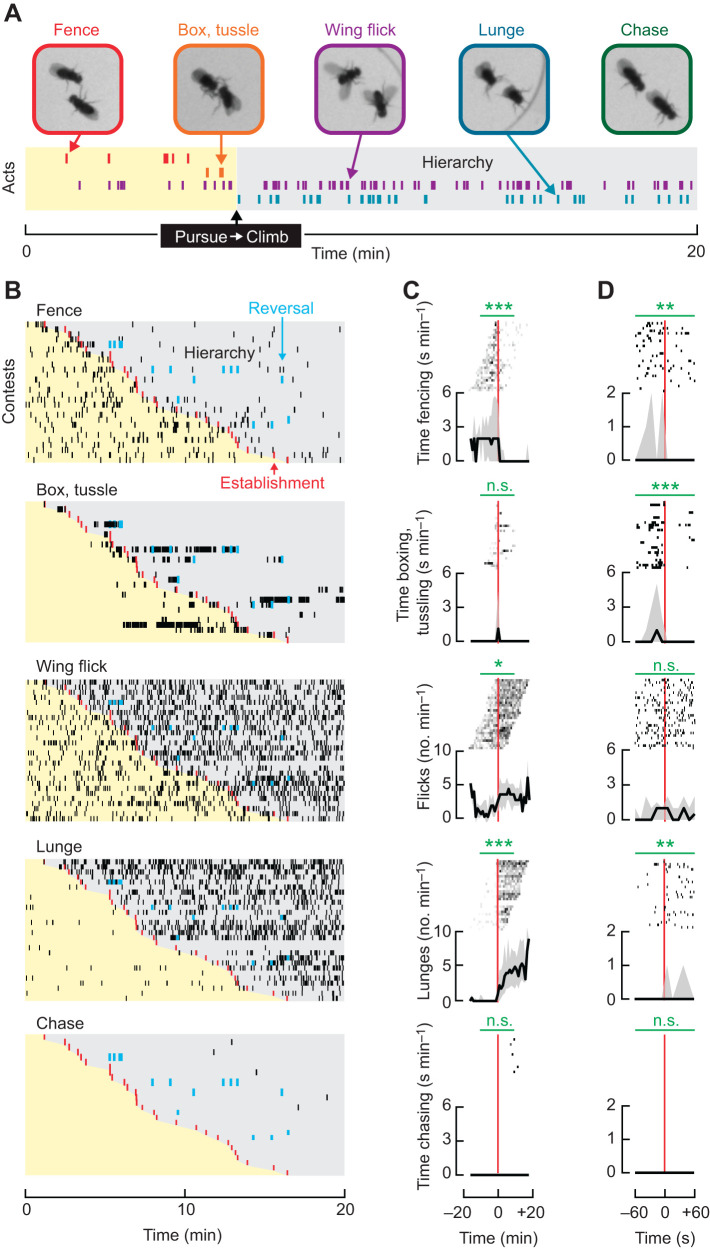
**Fence, box and tussle interactions precede the establishment of social hierarchy, whereas the majority of wing flicks, lunges and chases follow.** (A) Raster plot from a single contest illustrates the relationship between the observed reproducible sequence of behaviors and the establishment of social hierarchy. Each row and color corresponds to a different act. (B) Raster plots of examined behaviors displayed from early to late in sequence (top to bottom) show the temporal structure of discrete acts (black ticks). Within each plot, individual contests between pairs of naive males are ordered as rows by latency to the onset of establishment (red ticks). After establishment, males exhibit clear hierarchical relationships (gray shading), with occasional reversals in dominance (blue ticks). (C,D) Peri-event plots corresponding to examined behaviors aligned to the onset of establishment (vertical red line) with acts displayed above associated collective medians (black line) and interquartile ranges (gray envelope). (C) Entire contests with acts binned into 1-min intervals. (D) ±1-min windows with discrete acts. (C,D) Statistical comparisons for the fraction of time behaving or frequency of acts each minute within 5-min (C) and 1-min (D) windows are noted above plots (horizontal green lines). In all cases, the Wilcoxon signed rank test was used to assess whether differences in the observed acts exist between paired, equal-length windows of time before and after the establishment of social hierarchy. See Fig. S2C for details regarding data exclusion. Lunging events in current figure were automatically classified and are displayed without manual correction (see Materials and Methods). n.s., not significant; *P<0.05; **P<0.01; ***P<0.001.

From both individual contests and aggregate data, we observed reproducible patterns of behavior (Fig. 2). Males consistently displayed the highest levels of ‘fence’, from the beginning of contests until the onset of dominance. Thereafter, we observed only sporadic displays of ‘fence’ with no clear temporal structure. In contrast, ‘box, tussle’ appeared abruptly, peaked and then sharply decreased immediately prior to the onset of dominance (Fig. 2D), and on occasion also transiently reappeared preceding subsequent reversals in social status. ‘Wing flick’ was observed broadly, first occurring with low frequency at the beginning of contests and then making a salient uptick near the onset of dominance, with males continuing this elevated level of ‘wing flick’ throughout the remainder of the contest. Unexpectedly, ‘lunge’ was rarely observed before the establishment of dominance, but began and continually increased in intensity thereafter. Occurrences of ‘chase’ were never observed prior to dominance and emerged well after its establishment (see analyses associated with Table S1; as reported previously in Chen et al., 2002). Together, by aligning recordings from individual contests to the onset of social dominance, we have exposed which acts may causally relate to its establishment and those apparently more related to its maintenance.

### Lunges are insufficient for explaining hierarchical outcomes

The act of lunging is commonly used for studying aggression (see reviews by Hoopfer, 2016; Kravitz and Fernandez, 2015), with several studies suggesting they play a critical role in establishing dominance (Alekseyenko et al., 2010; Penn et al., 2010; Yurkovic et al., 2006). We, however, observed lunging almost always after the establishment of dominance (as seen in Fig. 2). To confirm this finding, we ran a second, independent group of contests and reviewed the incidences of lunging by direct inspection with slow playback to correct for false negative and semi-automatically for false positives displays (see Materials and Methods). After careful review, it was clear that males nearly exclusively lunged after the establishment of dominance, with almost all of the lunges executed by the dominant male (Fig. S3).

**Fig. 3.**
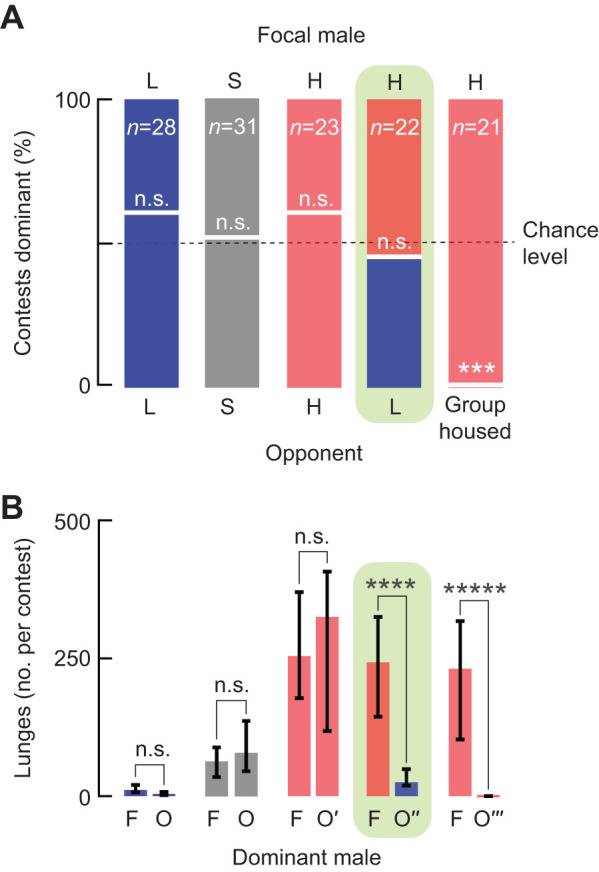
**Number of lunges executed by dominant individuals is insufficient for explaining hierarchical outcomes.** (A) Focal males (above) and opponents (below) were paired and the percentage of contests in which males became dominant are shown within each stacked bar chart. Focal males from genotypically low (L, blue), standard (S, gray) and high (H, light red) lunging lines became dominant at chance levels when paired with same-genotype opponents. For the tested pairing between mixed high and low lunging lines, males also became dominant in comparable amounts (shaded in green). In contrast, for contests in which high-lunging focal males were paired with socialized opponents (group housed) from the same genotype, focal males became dominant 100% of the time (compared with chance levels; Fisher’s exact, *P*=0.0005; right-most bar). In all cases, males were socially isolated unless noted. (B) Medians with interquartile ranges for the total number of lunges executed by dominant individuals from the contests reported in A above. The number of lunges executed by dominant focal males (F) from low (blue), standard (gray) and high (light red) lunging lines were no different than when those of paired opponents (O) from the same genotypes became dominant. Focal males from a high-lunging line (light red) displayed a comparable, high level of lunging when paired with all opponents (from left to right): males from the same genotype (O′, light red), genetically low-lunging males (O″, blue) or socialized males (O‴, light red) from the same genotype (Kruskal–Wallis, *H*_3_=5.92, *P*=0.1142). However, in tested pairings, the levels of lunges executed by the dominant, high-lunging focal males (light red) were significantly greater than those of dominant, low-lunging opponents (O″, blue; Wilcoxon rank-sum, *Z*=3.9244, *P*=0.00009; shaded in green) and socialized opponents from the same high-lunging line (O‴, light red; Wilcoxon sign, *P*<0.00001). The designation of ‘focal’ is independent of which male's wing was clipped for identification (see Materials and Methods), with the sole purpose of clarifying experiments. n.s., not significant; ***P<0.001; ****P<0.0001; *****P<0.00001.

From the repeated observation that lunging occurs after the onset of dominance, we reasoned that lunges were unlikely to play a role in its establishment. If this were true, then the number of lunges executed by a male over the 20-min contest would be unrelated to whether an individual emerges dominant. To test this, we set up contests with mixed pairs of males from identified genotypically high- and low-lunging lines (see Materials and Methods) and measured the total number of lunges executed by those males that emerged dominant. Males from genotypically high- and low-lunging lines became dominant at chance levels regardless of their opponents' genotypes (Fig. 3A). Moreover, we saw no influence of the total number of lunges on dominance outcomes, even when males from mixed pairs executed dramatically lopsided amounts of lunging (see fourth column in Fig. 3B, Fig. S1B,C for additional examples and Table S1 for details regarding genetic lines). It was therefore unsurprising to observe that males from both the high- and low-lunging genotypes executed the majority of lunges following the establishment of dominance (Fig. S4). Taken together, the results that males nearly exclusively lunge after the establishment of dominance and that the number of lunges executed by dominant males is unpredictive of social outcomes provide evidence that lunges do not establish dominance, and more likely play a role in its maintenance.

### Males express dominance by lunging at both familiar and unfamiliar opponents

For the majority of contests, we observed that only a single, stable hierarchical relationship formed between pairs of naive males (as shown in Fig. 2 and Fig. S3). One in five contests, however, included subsequent reversals in social status after the initial establishment of dominance, with lunges, sometimes by both males, clustering near each reversal. To better understand how these lunges relate to reversals in status, we verified which individual executed each lunge (see Materials and Methods). As in contests with only a single establishment of dominance, males nearly exclusively lunged after the initial establishment of dominance. Thereafter, currently and previously dominant males displayed the majority of lunges preceding reversals, and only dominant males lunged following the final reversal in status (Fig. S3). This analysis provides evidence that lunging is a good indicator of dominance status, and supports the interpretation that through subsequent reversals in dominance, lunges play a role in maintaining a male's social status (also see shaded green box in Fig. S5C–E).

**Fig. 4.**
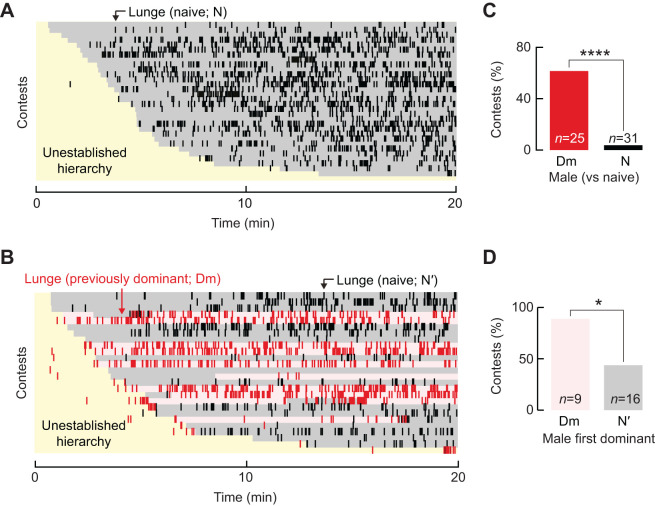
**Lunging persists as previously dominant males confront unfamiliar opponents in unexplored arenas.** (A,B) Raster plots show the temporal structure of lunges executed by naive (black ticks) and previously dominant (red ticks) males in relation to establishing social hierarchy. Within each plot, individual contests between pairs of males are ordered as rows by latency to the onset of establishment. Periods preceding establishment (yellow shading), and the prevailing dominance status for naive (gray shading) and previously dominant (light red shading) males are noted as observed within 20-min contests. (A) Pairs of naive males. (B) Previously dominant males paired with naive opponents in a second, unexplored arena. (C) The number of contests in which the previously dominant males (red bar; Dm) lunged prior to asserting dominance over naive males was significantly greater than the number of contests in which randomly selected individuals from pairs of naive males (black bar; N) lunged prior to establishing dominance (Chi-square: χ^2^_1_,_56_=16.07, *P=*0.00006). (D) The number of contests in which previously dominant males (light red bar; Dm) lunged prior to asserting dominant was also significantly greater than the number of contests in which they lunged early, failed and became subordinate to naive males (gray bar; N′; Chi-square: χ^2^_1,25_=4.89, *P*=0.02701). *P<0.05; ****P<0.0001.

In the experiments described above, both increased total time holding dominance and also the occurrence of reversals in social status contributed to a persistent high level of lunging by individuals against familiar opponents. Moreover, it has been reported that ‘winner’ males lunged and became dominant earlier in contests against naive opponents (Kim et al., 2018; Trannoy et al., 2016), and also, that males appeared to change their fighting tactics as a consequence of winning or losing (Trannoy and Kravitz, 2017). Therefore, to test whether lunging functions as a general tactic for maintaining dominance irrespective of opponent or location, we paired the emergent dominant males from a first contest between two naive males (Fig. 4A) with naive males in unexplored arenas (Fig. 4B). The most salient feature of these second contests was that the majority of previously dominant males lunged prior to establishing dominance (Fig. 4B,C), although their paired naive opponents did not. This pattern of behaviors was unlike that of pairs of naive males, which rarely lunged before the establishment of dominance (Fig. 4A,C). The temporal sequence for all other aggressive acts studied was comparable to that observed in contests between two naive males (data not shown). Finally, to examine if the precocious lunging helped previously dominant males assert dominance against unfamiliar males in unexplored arenas, we quantified the number of contests in which the previously dominant males lunged prior to asserting dominance. For the majority of these contests, previously dominant males successfully asserted their dominance (Fig. 4D). Taken together, these results support the understanding of lunging as a general expression of dominance that aids in maintaining a male's social status.

## DISCUSSION

Social dominance forms when individuals yield to the agonistic advances of others, often in the context of conflict over resources such as access to food, territories and reproductive opportunities (Drews, 1993). The relationship between specific aggressive acts and the establishment of social hierarchies remains contentious largely for they appear so highly correlated, even with disagreements over which drives the other (Francis, 1988). In this work, we use an explicit approach to characterize with high temporal resolution how a range of known acts of aggression relate to the establishment, maintenance and subsequent reversals in dominant hierarchical status.

The ‘pursue-to-climb’ criterion introduced within this work has several advantages. It is easy to describe and straightforward to implement. It accommodates the facts that adversaries compete over resources and includes an operationally defined ‘escape’, both of which are important ethological considerations (Chen et al., 2002; Hoffmann, 1987). Further, it provides the means to align and thus compare the onset of dominance, which varies in time across contests owing to differences in various influences (e.g. inherited factors, past social histories, current internal states, handling by the experimenter, environmental conditions and feedback between adversaries). Lastly, it can be used to update the classification of dominance if and when reversals in status occur after an initial hierarchical relationship has been established.

**Fig. 5.**
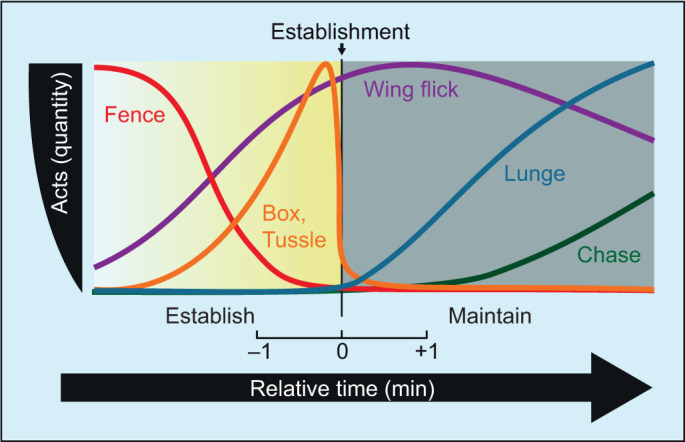
**Sequence of aggressive acts during the establishment of social hierarchy.** After adjusting to the experimental arena, pairs of males typically recognize each other as opponents. Successively, they then exhibit fencing (red line) and display low levels of wing flicks (purple line). As contests escalate (graded yellow box), fencing bouts decrease in frequency and are replaced by boxing and tussling (orange line) concomitantly with an increasing number of wing flicks. Transiently the amount of boxing and tussling peaks and then sharply drops immediately preceding the establishment of dominance (time=0). Lunging (blue line) follows the establishment of dominance, is the principal aggressive act during the maintenance of dominance (gray box), and is accompanied by a steady display of wing flicks. Finally, increased chasing (green line) observed after some time suggests stabilized hierarchical outcomes.

A significant challenge to studying complex social behaviors such as those related to the establishment of social hierarchy is that the numerous interactions possibly regulating them are often highly correlated with outcomes. By using a criterion to identify the establishment of dominance that is independent of the particular behaviors studied, we avoid a circular definition of social hierarchy, and thus allow for stronger claims of causality between specific aggressive acts and its emergence (for a graphical summary, see Fig. 5).

Unexpectedly, we show that almost always, only once naive males establish (or have held) dominance do they lunge, and moreover, they continue lunging against familiar opponents even after a dominance hierarchy is settled (as has been described previously (Chouhan et al., 2017; Yurkovic et al., 2006)). From this and our observation that previously dominant males precociously lunge and successfully assert dominance over unfamiliar males in novel settings, we propose that lunging is likely uninvolved in the establishment of dominance, but rather symptomatic of a changed internal state (Hoopfer et al., 2015) and plays a role in maintaining an individual's social status.

The complexity of reversals in dominance requires more study. In an attempt to more fully understand the role that the examined aggressive acts played in the reversal of hierarchical status, we analyzed a larger number of contests that included reversals. From a preliminary characterization of these contests, lunges appeared as just one of several interacting acts likely involved in this process (Fig. S5). It is clear, as with the initial establishment of dominance, that further consideration is needed to formally conclude which acts of aggression causally drive these dynamic, reversing and escalating exchanges.

In natural settings, flies rarely exist as isolated pairs. Thus, the atypical social grouping examined within this work motivates consideration of how the interactions observed between pairs of males might contribute to the emergent social dynamics in larger groups (e.g. despotic ‘winner takes all’, linear ‘pecking order’ or dominance transitivities). Although it was beyond our intent to address the emergent of social hierarchy within larger groups, our observation that previously dominant males precociously lunge at unfamiliar opponents in novel settings supports the notion that dominance is an individual-centric behavioral state that does not necessitate past experience with a particular opponent or with a recognized location. And it is noteworthy that this understanding alone can explain the various reports of winner and loser effects (Kim et al., 2018; Penn et al., 2010; Trannoy et al., 2015, 2016; Vrontou et al., 2006; Yurkovic et al., 2006). However, it is also important to point out that our observations do not exclude the superposition of additional influences. Therefore, at this time, what drives the structure and hierarchy formation in larger groups remains an open question.

In summary, we demonstrate that identifying biological meaningful epochs of time with a criterion that is independent of precise descriptions of behavior helps in establishing both nuanced and holistic understandings of this complex behavioral phenotype. These understandings should assist in refining the roles of known acts of aggression (Chen et al., 2002; Duistermars et al., 2018; Jonsson et al., 2011), and possibly discovering new ones. Finally, when used in conjunction with high-throughput screens, the approach and work described herein provide a framework to clarify the genetic and neural mechanisms underlying the coordination of behaviors involved in establishing hierarchical social relationships in insects.

## Supplementary Material

Supplementary information

## References

[JEB232439C1] Alekseyenko O. V., Lee C. and Kravitz E. A. (2010). Targeted manipulation of serotonergic neurotransmission affects the escalation of aggression in adult male *Drosophila melanogaster*. *PLoS ONE* 5, e10806 10.1371/journal.pone.0010806 20520823 PMC2875409

[JEB232439C2] Anderson D. J. (2016). Circuit modules linking internal states and social behaviour in flies and mice. *Nat. Rev. Neurosci.* 17, 692-704. 10.1038/nrn.2016.125 27752072

[JEB232439C3] Asahina K. (2017). Neuromodulation and strategic action choice in *Drosophila* aggression. *Annu. Rev. Neurosci.* 40, 51-75. 10.1146/annurev-neuro-072116-031240 28375770

[JEB232439C4] Baier A., Wittek B. and Brembs B. (2002). *Drosophila* as a new model organism for the neurobiology of aggression? *J. Exp. Biol.* 205, 1233-1240. 11948200 10.1242/jeb.205.9.1233

[JEB232439C5] Berridge K. C. (2004). Motivation concepts in behavioral neuroscience. *Physiol. Behav.* 81, 179-209. 10.1016/j.physbeh.2004.02.004 15159167

[JEB232439C6] Certel S. J. and Kravitz E. A. (2012). Scoring and analyzing aggression in *Drosophila*. *Cold Spring Harb. Protoc.* 2012, 319-325. 10.1101/pdb.prot068130 22383642

[JEB232439C7] Chen P. and Hong W. (2018). Neural circuit mechanisms of social behavior. *Neuron* 98, 16-30. 10.1016/j.neuron.2018.02.026 29621486 PMC6028944

[JEB232439C8] Chen S., Lee A. Y., Bowens N. M., Huber R. and Kravitz E. A. (2002). Fighting fruit flies: a model system for the study of aggression. *Proc. Natl. Acad. Sci. USA* 99, 5664-5668. 10.1073/pnas.082102599 11960020 PMC122828

[JEB232439C9] Chouhan N. S., Mohan K. and Ghose A. (2017). cAMP signaling mediates behavioral flexibility and consolidation of social status in *Drosophila* aggression. *J. Exp. Biol.* 220, 4502-4514. 10.1242/jeb.165811 28993465

[JEB232439C10] Dankert H., Wang L., Hoopfer E. D., Anderson D. J. and Perona P. (2009). Automated monitoring and analysis of social behavior in *Drosophila*. *Nat. Methods* 6, 297-303. 10.1038/nmeth.1310 19270697 PMC2679418

[JEB232439C11] Dierick H. A. (2007). A method for quantifying aggression in male *Drosophila melanogaster*. *Nat. Protoc.* 2, 2712-2718. 10.1038/nprot.2007.404 18007606

[JEB232439C12] Drews C. (1993). The concept and definition of dominance in animal behaviour. *Behaviour* 125, 283-313. 10.1163/156853993X00290

[JEB232439C13] Duistermars B. J., Pfeiffer B. D., Hoopfer E. D. and Anderson D. J. (2018). A brain module for scalable control of complex, multi-motor threat displays. *Neuron* 100, 1474-1490.e4. 10.1016/j.neuron.2018.10.027 30415997 PMC6314657

[JEB232439C14] Francis R. C. (1988). On the relationship between aggression and social dominance. *Ethology* 78, 223-237. 10.1111/j.1439-0310.1988.tb00233.x

[JEB232439C15] Freudenberg F., Carreño Gutierrez H., Post A. M., Reif A. and Norton W. H. J. (2015). Aggression in non-human vertebrates: genetic mechanisms and molecular pathways. *Am. J. Med. Genet. Part B Neuropsychiatr. Genet.* 171, 603-640. 10.1002/ajmg.b.32358 26284957

[JEB232439C17] Haegorn J., Hailpern J. and Karahalios K. G. (2008). VCode and VData: illustrating a new framework for supporting the video annotation workflow. *Extended Abstr. AVI*, 317-321. 10.1145/1385569.1385622

[JEB232439C18] Hoffmann A. A. (1987). A laboratory study of male territoriality in the sibling species *Drosophila melanogaster* and *D. simulans*. *Anim. Behav.* 35, 807-818. 10.1016/S0003-3472(87)80117-3

[JEB232439C19] Hoffmann A. A. (1990). The influence of age and experience with conspecifics on territorial behavior in *Drosophila melanogaster*. *J. Insect Behav.* 3, 1-12. 10.1007/BF01049191

[JEB232439C20] Hoopfer E. D. (2016). Neural control of aggression in *Drosophila*. *Curr. Opin. Neurobiol.* 38, 109-118. 10.1016/j.conb.2016.04.007 27179788

[JEB232439C21] Hoopfer E. D., Jung Y., Inagaki H. K., Rubin G. M. and Anderson D. J. (2015). P1 interneurons promote a persistent internal state that enhances inter-male aggression in *Drosophila*. *eLife* 4, e11346 10.7554/eLife.11346 26714106 PMC4749567

[JEB232439C22] Jonsson T., Kravitz E. A. and Heinrich R. (2011). Sound production during agonistic behavior of male *Drosophila melanogaster*. *Fly* 5, 29-38. 10.4161/fly.5.1.13713 20953152 PMC3052870

[JEB232439C23] Kim Y.-K., Saver M., Simon J., Kent C. F., Shao L., Eddison M., Agrawal P., Texada M., Truman J. W. and Heberlein U. (2018). Repetitive aggressive encounters generate a long-lasting internal state in *Drosophila melanogaster* males. *Proc. Natl. Acad. Sci. USA* 115, 1099-1104. 10.1073/pnas.1716612115 29339481 PMC5798363

[JEB232439C24] Kravitz E. A. and Fernandez M. d. I. P. (2015). Aggression in *Drosophila*. *Behav. Neurosci.* 129, 549-563. 10.1037/bne0000089 26348714

[JEB232439C25] Kravitz E. A. and Huber R. (2003). Aggression in invertebrates. *Curr. Opin. Neurobiol.* 13, 736-743. 10.1016/j.conb.2003.10.003 14662376

[JEB232439C26] Penn J. K. M., Zito M. F. and Kravitz E. A. (2010). A single social defeat reduces aggression in a highly aggressive strain of *Drosophila*. *Proc. Natl. Acad. Sci. USA* 107, 12682-12686. 10.1073/pnas.1007016107 20616023 PMC2906583

[JEB232439C27] Sherer L. M. and Certel S. J. (2019). The fight to understand fighting: neurogenetic approaches to the study of aggression in insects. *Curr. Opin. Insect Sci.* 36, 18-24. 10.1016/j.cois.2019.06.004 31302354 PMC6906251

[JEB232439C28] Trannoy S. and Kravitz E. A. (2017). Strategy changes in subsequent fights as consequences of winning and losing in fruit fly fights. *Fly* 11, 129-138. 10.1080/19336934.2016.1259041 27834611 PMC5406166

[JEB232439C29] Trannoy S., Chowdhury B. and Kravitz E. A. (2015). Handling alters aggression and “loser” effect formation in *Drosophila melanogaster*. *Learn. Mem.* 22, 64-68. 10.1101/lm.036418.114 25593291 PMC4341365

[JEB232439C30] Trannoy S., Penn J., Lucey K., Popovic D. and Kravitz E. A. (2016). Short and long-lasting behavioral consequences of agonistic encounters between male *Drosophila melanogaster*. *Proc. Natl. Acad. Sci. USA* 113, 4818-4823. 10.1073/pnas.1520953113 27071097 PMC4855558

[JEB232439C31] Vrontou E., Nilsen S. P., Demir E., Kravitz E. A. and Dickson B. J. (2006). fruitless regulates aggression and dominance in *Drosophila*. *Nat. Neurosci.* 9, 1469-1471. 10.1038/nn1809 17115036

[JEB232439C32] Wang L., Dankert H., Perona P. and Anderson D. J. (2008). A common genetic target for environmental and heritable influences on aggressiveness in *Drosophila*. *Proc. Natl. Acad. Sci. USA* 105, 5657-5663. 10.1073/pnas.0801327105 18408154 PMC2311352

[JEB232439C33] Yurkovic A., Wang O., Basu A. C. and Kravitz E. A. (2006). Learning and memory associated with aggression in *Drosophila melanogaster*. *Proc. Natl. Acad. Sci. USA* 103, 17519-17524. 10.1073/pnas.0608211103 17088536 PMC1634832

