# Supplementary material for: Social hierarchy is established and maintained with distinct acts of aggression in male Drosophila melanogaster
Source: J Exp Biol. 2020 Dec 23;223(24):jeb232439. doi: 10.1242/jeb.232439 (PMC7774903; doi:10.1242/jeb.232439)
Supplement: Supplementary information [file jexbio-223-232439-s1.pdf]

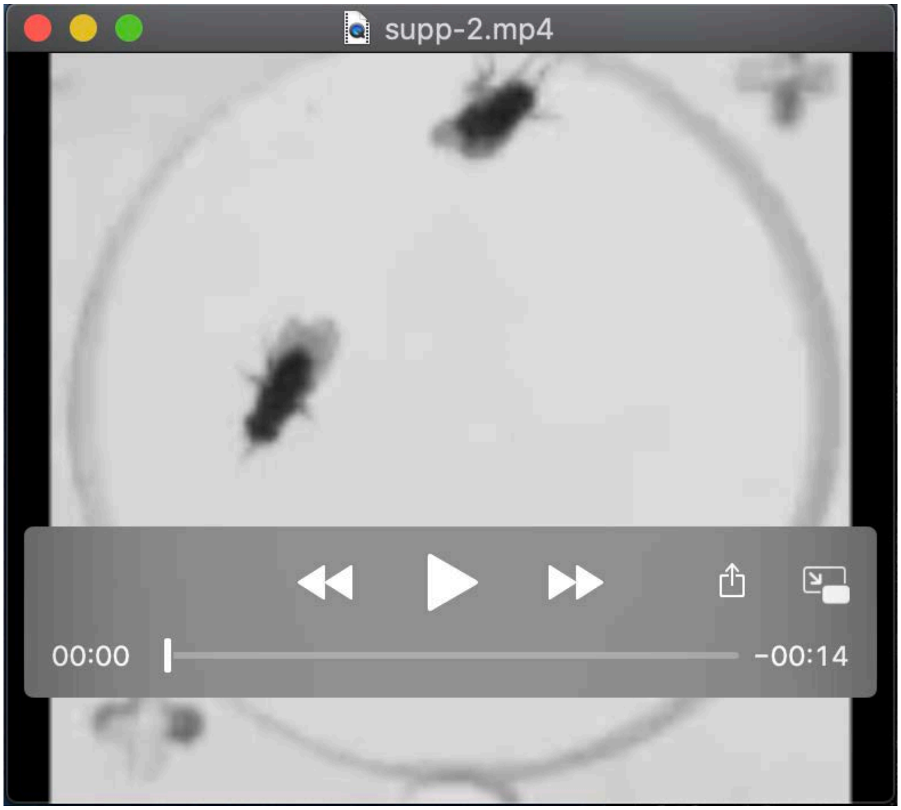

Movie 1. Established hierarchical relationship. Example movie from a 20-minute contest showing an established hierarchical relationship, wherein a subordinate male repeatedly attempts to climb the wall of the arena while a dominant male (clipped wing) guards the floor.

SUPPLEMENTAL FIGURES

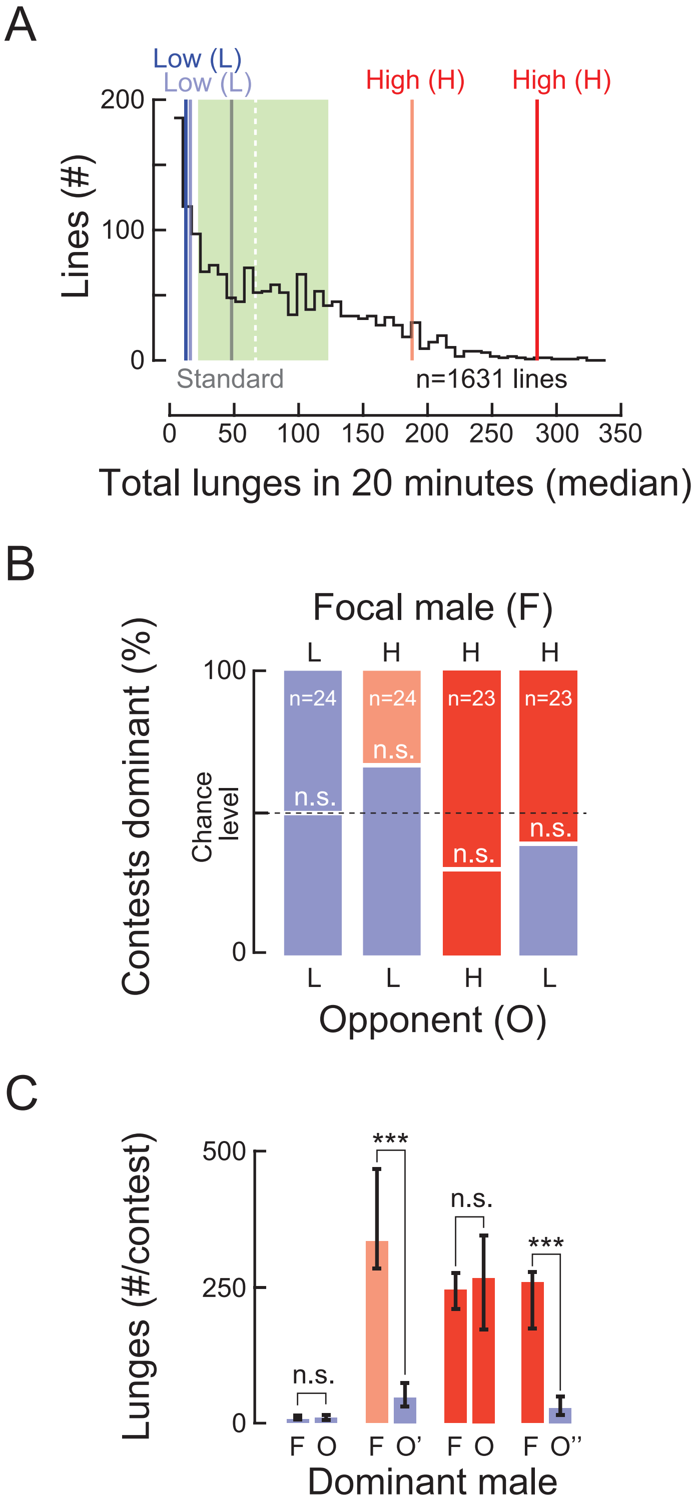

Journal of Experimental Biology • Supplementary information

Fig. S1. Independently isolated genotypically low, standard, and high lunging lines identified from a P-element screen; additional genotypically high and low lunging lines corroborate findings in Fig. 3. (A) The distribution of P-element lines screened before outcrossing is skewed right (black, stair-step line) with the median (vertical, white dashed line) and interquartile range (green shading) centered around 67 total lunges, as calculated by summing the total lunges executed by pairs of same-genotype males from 20-minute contests. The median number of lunges executed by the genotypic standard (gray) used throughout the study and also the low (blue, light blue) and high (light red, red) lines are shown after outcross. (B) Focal males (F, above) and opponents (O, below) were paired in various combinations and the percentage of contests in which males became dominant are shown within each stacked bar chart, with adversaries coded by letter and color as indicated in “A” above. Focal males became dominant at chance levels in all contests. (C) Lunging executed by dominant males in both same- and mixed-genotype pairings were consistent with those reported in Fig. 3. Dominant focal or opponent males arising from same-genotype pairs displayed equivalent amounts of lunges (F vs. O, light blue and red). Focal males from an additional high-lunging line (F, red) displayed a comparable, high level of lunging regardless of opponent (Kruskal-Wallis test; H (1)=0.0039, $p=0.9503$). However, in tested pairings the levels of lunges executed by both the original and the additional dominant, high-lunging focal males (F, light red; F, red) were significantly greater than an additional dominant, low-lunging opponent (vs. O’, light blue; Wilcoxon Rank-Sum, Z=3.8884, $p=.00010$ and versus O’’, light blue; Wilcoxon Rank-Sum, Z=3.8111, $p=.00014$).

Journal of Experimental Biology • Supplementary information

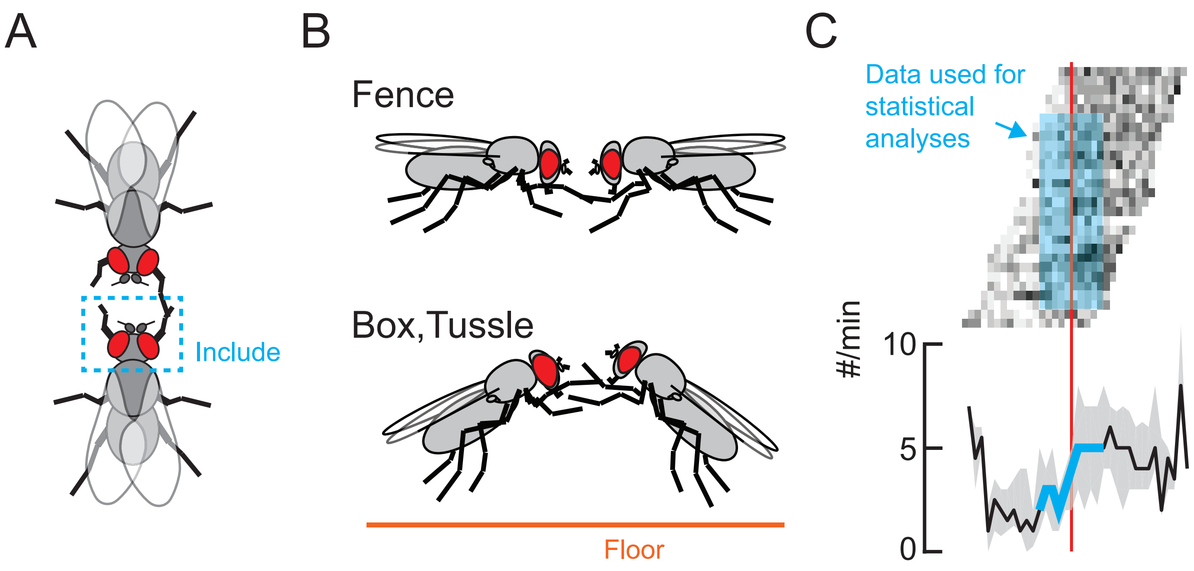

Fig. S2. Details for including and classifying "Fence" versus "Box, Tussle" interactions; example data to show the requisite exclusions for balanced statistical analyses. (A) Illustration drawn from a top viewing angle to clarify the criteria for inclusion. For an interaction to be included the focal male must touch the front legs or head of the opponent (blue dashed box). (B) Side views showing the postural difference between "Fence" and "Box, Tussle." Interactions that included touch from a prone stance were classified as "Fence." Any touching when one or both males exhibited an erect posture with both front legs lifted off the floor were classified as "Box, Tussle." Interactions in which males disengaged, directly executed a 360-degree rotational turn, and then reengaged were annotated as a single continuous act. (C) An example dataset shows the data used for statistical analyses. In order to apply paired statistical tests, only contests allowing five minutes or more (always equal) windows of time before and after the establishment of social hierarchy were used unless otherwise noted. In the example shown only contests with five minutes of recorded data before and after establishment of dominance were used (blue shaded box, above; median frequency of behavior for corresponding period denoted as bold, blue line, below). In this example, data recorded earlier than five minutes preceding and following establishment and also the data from the entire first five and last two contests were excluded. The exclusions decreased the sample size, and thereby the statistical significance, yet never qualitatively changed results.

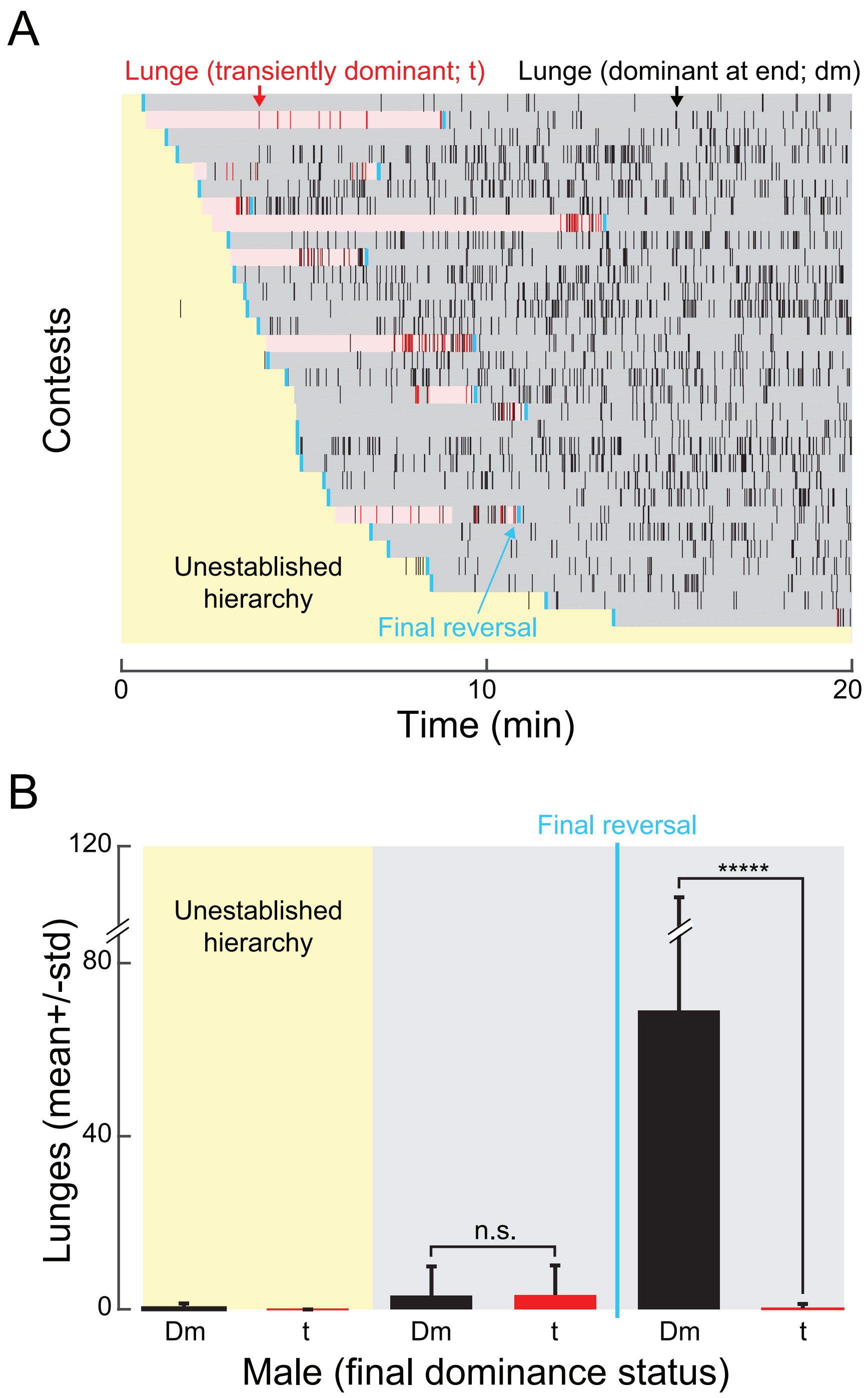

Fig. S3. Males express dominance by lunging. (A) Raster plot displaying the temporal structure of lunges executed by transiently dominant males (red ticks; t) and those observed dominant at end (black ticks; dm) in relation to the initial establishment and reversals in social dominance. Individual contests between pairs of naïve males are

ordered as rows by latency to the onset of establishment. Periods preceding establishment (yellow shading), ultimately transient (light red shading), and of final dominance status (gray shading) are noted as observed within 20-minute contests. Final reversals in social status for each contest are indicated with blue ticks (ticks for intermediate reversals were excluded for clarity). (B) Lunging reflects dominance. Uncommonly, lunges were observed before the initial establishment of dominance (bar plots highlighted by yellow shading). In the period following the initial establishment yet before final reversals in dominance (vertical, blue line), currently and previously dominant males executed the majority of the lunges in comparable, low amounts irrespective of final dominance outcomes. Thereafter, all lunges came from dominant males (Dm, black bar), and subordinate males (t, red bar) displayed none (Wilcoxon rank sum, Z=7.1057, $p<.00001$). This figure includes the identities of which male lunged and also denotes any reversals in social status from contests between the pairs of naïve males reported in Fig. 4A.

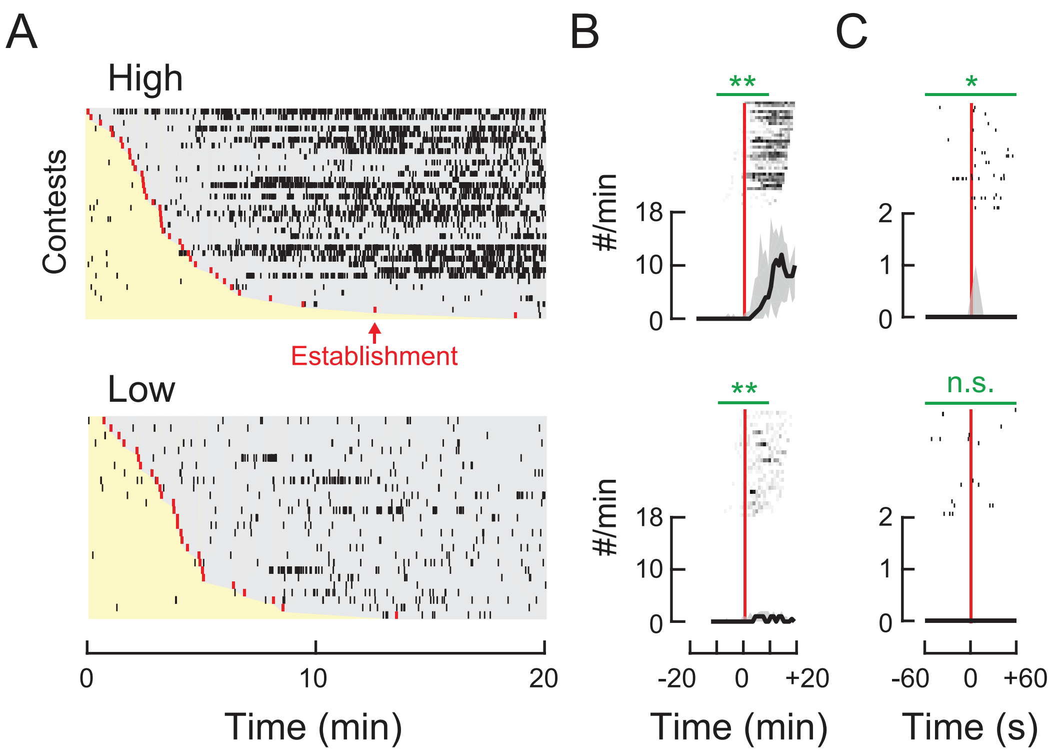

Fig. S4. Lunges were executed after establishing dominance irrespective of genotype. (A) Raster plots show the temporal structure for lunges executed by males from genotypically high (top) and low (bottom) lunging lines (lunge = black tick). Within each plot, individual contests between pairs of naïve males from the same genotype (high vs. high and low vs. low) are ordered as rows by latency to the onset of establishment (red ticks). After the establishment of dominance males exhibit clear hierarchical relationships (gray shading). (B,C) Corresponding peri-event plots aligned to the onset of establishment (vertical, red line) with lunges displayed above associated collective medians (black line) and interquartile ranges (gray envelope). (B) Entire contests with lunges from each contest binned into one-minute intervals. (C) ±one-minute windows with distinct lunging events. (B,C) Statistical comparisons for the frequency of lunges within five- (B) and one-minute (C) windows prior to and following establishment are noted above plots (horizontal, green lines). In all cases the Wilcoxon signed rank test was used as described in Fig. S2C. Lunging events in current figure were automatically classified and are displayed without manual correction (see Methods).

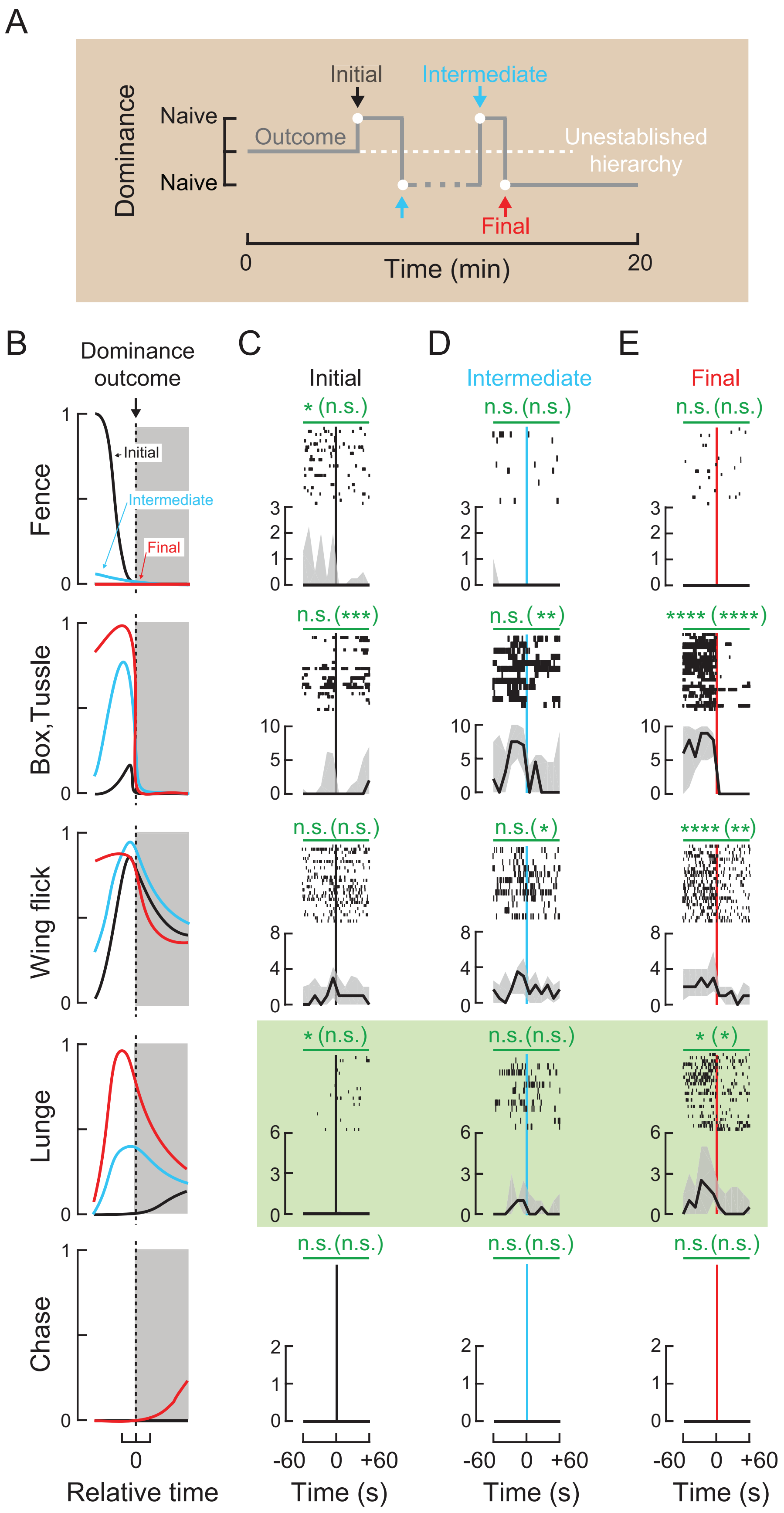

Journal of Experimental Biology • Supplementary information

Fig. S5. Recurring aggressive acts escalate through subsequent reversals in dominance. (A) Schematic illustrating the establishment and subsequent reversals in dominant hierarchical status in contests between pairs of naïve males. The gray, switching line indicates the state of dominance with colored arrows indicating the onset (white dot) for the initial establishment (Initial, black), subsequent reversals (Intermediate, blue), and the final hierarchical outcome (Final, red). (B) Idealized results for the observed changes of aggressive acts among the initial establishment (black line) and intermediate (blue line) and final (red line) reversals of dominance status (gray shaded box). A value of "1" indicates the highest level for any particular act to help clarify its relative increase or decrease; drawings are based on the data in Fig. 2, those reported here in "C-E," and contests using males from the genotypically high and low lunging lines (data not shown). Depicted in the top panel of "B," early on males exhibit the highest levels of "Fence," which then drops during the minute immediately preceding the initial establishment (black line). From then on only intermittent and low levels of fencing were observed through subsequent reversals (Intermediate, blue and Final, red lines). In contrast, as shown in the next panel down, the amount of "Box, Tussle" peaks during the minute immediately preceding the initial establishment (black line), and then peaks again and again, increasing and broadening through subsequent reversals (Intermediate, blue and Final, red lines). (C-E) Peri-event plots with ±one-minute windows aligned to the initial establishment (C; vertical, black line), the intermediate subsequent reversals (D; vertical, blue line), and final outcome (E; vertical, red line) of social hierarchy. Discrete acts are displayed above associated collective medians (black line) and interquartile ranges (gray envelope). (C-E) Statistical comparisons for the frequency of aggressive acts within ±one-minute and ±10-second (within parentheses) windows prior to and following establishment are noted above plots (horizontal, green lines). In all cases the Wilcoxon signed rank test was used as described in Fig. S2C. Lunges increased, formed clusters, and the peaks of clusters shifted forward in time relative to each subsequent reversal (C-E; shaded, green box). Lunging increased in a scalable manner (One-way ANOVA: df(2), F(22.5470), $p<.00001$ and followed by post hoc comparisons: Initial versus Intermediate, $p=.0135$, Intermediate versus Final, p=*.0475*, and Initial versus Final, $p<.00001$; all with Bonferroni correction). The shift in

Journal of Experimental Biology • Supplementary information

peaks was quantified as a procession index; PI = (Lunges *minute preceding* - Lunges *minute following*) / (Lunges *minute preceding* + Lunges *minute following*). One-way ANOVA: df(2), F(6.1421), *p=.0049* and followed by post hoc comparisons: Initial versus Intermediate, *p=n.s.*, Intermediate versus Final, *p=n.s.*, and Initial versus Final, *p=.0046*; all with Bonferroni correction). The intensity for several of the aggressive acts increased alongside that of lunges. Collectively, the characterization exposes a recurring structure that emerges during protracted engagements – one in which both males reciprocally escalate fights just prior to reversals in status – seemingly in attempt to maintain or reclaim social dominance. Data within this figure came from contests all containing reversals in dominance (n=25) identified from larger set of adversarial contests (n=146).

Journal of Experimental Biology • Supplementary information

SUPPLEMENTAL TABLE

Table S1. Behavioral measures for the out-crossed P-element lines used within manuscript. P-element lines ordered by row from fewest to greatest number of lunges (median ±quartile) per 20-minute contest as identified within screen after outcrossing. Line names indicate batch followed by trial number and come from the labelling scheme of our laboratory. Color and intensity may be used to associate lines with results from Fig. 3 and Fig. S1. In addition to its high levels of lunges, the line 9.3 was chosen for its high amounts of chase (see below). Consequently, to support the notion that chase events follow the establishment of social hierarchy as observed in Fig. 2, we analyzed chase events in contests between pairs of males from 9.3 and also in mixed contests between males from 9.3 and the those from the low-lunging line 11.261. In both cases, we observed that all chase events occurred following the establishment of hierarchy (compared to equal numbers of chase events before and after establishment; 9.3 versus 9.3, Fisher exact, n=18; *p=.0010*; 9.3 versus 11.261, Fisher exact, n=11; *p=.0351*). Together, combining the above results with those from Fig. 2, we report that we have only observed chases following the establishment social hierarchies, Fisher exact, n=34, *p<.00001*.

Line	Color	Intensity	Sample size (n=contests)	Tussle	Wing flick	Lunge	Chase
**11.261**	Blue	Low	62	1 (0/4.3)	66.5 (12/128)	12.5 (1/26)	0 (0/1)
**15.46**	Light blue	Low	64	1 (0/4)	66 (24.5/213.8)	16 (6.5/37.5)	0 (0/1)
**5.116**	Gray	Standard	301	3 (0/8)	95 (57/149)	48 (23.5/104)	0 (0/3)
**9.3**	Light red	High	24	2 (0.9/7.6)	138 (73/212.6)	184 (98.5/236)	4 (0/16)
**11.27**	Red	High	52	3 (1/8.3)	138 (85.9/214.9)	277 (186/391)	0 (0/2)

Journal of Experimental Biology • Supplementary information
